# Supplementary material for: Identification and functional analysis of non-coding regulatory small RNA FenSr3 in Bacillus amyloliquefaciens LPB-18
Source: PeerJ. 2023 May 15;11:e15236. doi: 10.7717/peerj.15236 (PMC10194069; doi:10.7717/peerj.15236)
Supplement: Supplemental Information 4 [file peerj-11-15236-s004.zip › KO/CK-vs-T1_map/map04974.html]

KEGG PATHWAY: Protein digestion and absorption - Reference pathway


|  |  |
| --- | --- |
| **Protein digestion and absorption - Reference pathway** |  |

[
Pathway menu
| Organism menu
| Pathway entry
| Show description
| User data mapping
]

Protein is a dietary component essential for nutritional homeostasis in humans. Normally, ingested protein undergoes a complex series of degradative processes following the action of gastric, pancreatic and small intestinal enzymes. The result of this proteolytic activity is a mixture of amino acids and small peptides. Amino acids (AAs) are transported into the enterocyte (intestinal epithelial cell) by a variety of AA transporters that are specific for cationic (basic) AA, neutral AA, and anionic (acidic) AA. Small peptides are absorbed into enterocytes by the PEPT1 transporter. Inside enterocytes peptides are hydrolyzed, and the resulting amino acids are released together with those absorbed by AA transporters into blood via multiple, basolateral, AA transporters. Hydrolysis-resistant peptides, however, are transported out of the cells by a basolateral peptide transporter that has not been identified molecularly.

|  |  |  |
| --- | --- | --- |
| Reference pathway | 184% 150% 122% 100% 82% 67% 55% | 图片下载 |
